# Origin and Evolution of H1N1/pdm2009: A Codon Usage Perspective

**DOI:** 10.3389/fmicb.2020.01615

**Published:** 2020-07-14

**Authors:** Fucheng Guo, Jinjin Yang, Junbin Pan, Xianghui Liang, Xuejuan Shen, David M. Irwin, Rui-Ai Chen, Yongyi Shen

**Affiliations:** ^1^College of Veterinary Medicine, South China Agricultural University, Guangzhou, China; ^2^Guangdong Laboratory for Lingnan Modern Agriculture, Guangzhou, China; ^3^Department of Laboratory Medicine and Pathobiology, University of Toronto, Toronto, ON, Canada; ^4^Banting and Best Diabetes Centre, University of Toronto, Toronto, ON, Canada; ^5^Guangdong Enterprise Key Laboratory of Biotechnology R&D of Veterinary Biological Products, Zhaoqing, China

**Keywords:** codon usage, H1N1/pdm2009, swine influenza virus, triple-reassortant swine viruses, Eurasian avian-like swine viruses, influenza A virus

## Abstract

The H1N1/pdm2009 virus is a new triple-reassortant virus. While Eurasian avian-like and triple-reassortant swine influenza viruses are the direct ancestors of H1N1/pdm2009, the classic swine influenza virus facilitate the spectrum of influenza A diversity in pig population when the reassortant events occurred during 1998 to April 2009. The factors that facilitate the final formation of this gene constellation for H1N1/pdm2009 virus from this complex gene pool remain unknown. Since a novel successful virus should efficiently replicate and transmit in their hosts, in this study, we estimated the adaptability of the codon usage patterns of the pool of genes from these lineages of swine influenza viruses to the human expression system. We found that the MP and NA genes of Eurasian avian-like swine influenza viruses, and the PB2, PB1 and PA genes of triple-reassortant swine influenza viruses were best adapted to the human codon usage pattern. As these genes participated in the development of H1N1/pdm2009, they might help in viral replication and strengthen its competitiveness during its emergence. After its emergence in the human population, a gradual optimization of codon usage patterns between 2009 and 2019 to the human codon usage for the H1N1/pdm2009 genes was detected. This reveals that ongoing adaptive evolution, after its original incursion, occurred to further increase the adaptability of overall gene cassette to human expression system.

## Introduction

The H1N1/pdm2009 virus was first isolated from humans in North America in April 2009 (Smith et al., 2009). After its emergence, the H1N1/pdm2009 virus has replaced the previous human seasonal H1N1 and has circulated as a seasonal virus, posing a substantial risk to human populations. This virus is the product of reassortments among multiple swine influenza virus lineages: its NA and M genes were derived from the Eurasian avian-like swine H1N1 influenza virus (EAsw SIV), while its other genes were from the triple-reassortant (TRsw) SIV with PB2 and PA derived from avian H1N1, PB1 from human H3N2, and HA, NP, NS, NA, and M from classical swine (Csw) H1N1 (Newman et al., 2008; Smith et al., 2009).

The EAsw H1N1, which is derived from avian H1N1, was first detected in Belgium in 1979 and since then has become established in the European swine population (Pensaert et al., 1981; Scholtissek et al., 1983; Brown, 2000). In North America, Csw H1N1 SIVs were the major cause of swine influenza since their initial isolation in 1930 up until 1998, when the TRsw H3N2 SIVs emerged (Newman et al., 2008). Co-circulation and mixing of the TRsw H3N2 SIVs with established swine lineages resulted in subsequent generation of new H1N1 and H1N2 reassortant swine viruses (Olsen, 2002). In Asia, Csw H1N1 SIVs viruses continue to cause endemics in southern China and Southeast Asia later than 2005, in addition to other identified viruses like human H3N2, EAsw H1N1 and North American TRsw SIVs (Qi et al., 2009).

These SIVs are poorly adapted to humans, with evidences that showed a substantially lower growth capacity and limited transmissibility than H1N1/pdm2009 *in vitro* and *ex vivo* cultures of the human respiratory tract, and only caused occasional human infections (Myers et al., 2007; Chan et al., 2011; Zhou et al., 2018). By contrast, H1N1/pdm2009 virus, derived from the gene pool of multiple coexisting lineages of swine influenza viruses, spreads rapidly and establishes sustained transmission in human populations, demonstrating its great adaptation to the human population. However, not only we are less clear on the temporospatial sequence of these reassortant events, but also the factors that facilitate such reassortments and eventually gave rise to the origin of this successful novel virus, is still unknown.

Multiple crucial factors within the virus and the host such as receptor-binding specificity and affinity, host-specific immune responses, are assumed to be the key players of viral adaptation in host system (Long et al., 2019). In addition, viral codon usage patterns significantly govern their replication and fitness in host microenvironment (Carlini, 2004). Viruses depend on host translational machinery, and codon usage patterns that are better adapted to its host facilitate the efficiency and accuracy of protein production at multiple levels while maintaining amino acid sequence (Tian et al., 2018). It has been reported that modern H3N2 viruses are translated more efficiently due to their acquisition of codon usage patterns that better reflect tRNA availability in IFN-treated cells (Smith et al., 2018). Optimizing or de-optimizing codon usage patterns of parasitic viruses, according to the codon usage patterns of specific hosts, has a significant impact on the replication of viruses in their hosts (Mueller et al., 2006, 2010; Coleman et al., 2008).

The gene constellation of triple-reassortant H1N1/pdm2009 is derived from the gene pool of multiple coexisting swine influenza viruses, where advantageous segments can aggregate to develop a novel virus. As influenza viruses depend on cellular functions and factors for their own propagation, a better match with host’s tRNA pool should contribute to the efficient use of host resources and then the faster replication of the virus (Bera et al., 2017; Li et al., 2018; Luo et al., 2019b). Since successful novel viruses should efficiently replicate in their hosts, we speculate that during the origin of H1N1/pdm2009, the gradual aggregation of advantageous genes from multiple SIVs lineages that better fit the codon usage of human, which benefit its efficient replication, led to the outbreak of the virus. The SIVs pertaining to EAsw, TRsw and Csw lineages, which directly or indirectly contributed to the gene constellation of H1N1/pdm2009, have sustained circulation for more than 10 years, accounting for the majority of complex spectrum of influenza virus diversity in pig population, and maintained endemics in pig population during 1998 to 2009. Therefore, in this study, we compared the codon usage of these lineages of SIVs to study the selection strategies of H1N1/pdm2009. In addition, novel viruses often undergo adaptive evolution to accumulate genetic changes to become more adapted to their host, thus we investigated the adaptation of codon usage of the H1N1/pdm2009 since its emergence.

## Materials and Methods

### Data Source and Preliminary Treatment

All influenza sequences used in this study were downloaded from the Influenza Virus Resource at the National Center for Biotechnology Information (NCBI)^1^, and the Global Initiative on Sharing Avian Influenza Data^2^. Redundant sequences, laboratory strains and short sequences (<85% of the corresponding gene) were removed.

To identify the complex spectrum of influenza virus diversity in pig population and explore the history of the origin of H1N1/pdm2009, we downloaded 320 (comprising of 188 H1N1, 66 H1N2, and 68 H3N2 genomes) complete swine-isolated influenza genomes collected from 1998 to April 2009 (Supplementary Table S1).

To study the evolution of H1N1/pdm2009 since its origin, we collected human-isolated H1N1/pdm2009 sequences from April 2009 to April 2019. For comparative analysis, contemporary human-isolated sequences pertaining to H3N2 were also included. For H1N1/pdm2009, the final dataset contained 20831, 14107, 17605, 14542, 14076, 15483, 15047, and 15320 unique sequences for segment 4 (HA), 7 (MP), 6 (NA), 5 (NP), 8 (NS), 3 (PA), 2 (PB1), and 1 (PB2), respectively (Supplementary Table S2). In case of H3N2, the dataset comprised of 11698, 5823, 9445, 6835, 5480, 7607, 7835, and 7913 unique sequences for segment 4 (HA), 7 (MP), 6 (NA), 5 (NP), 8 (NS), 3 (PA), 2 (PB1), and 1 (PB2), respectively (Supplementary Table S3).

Sequences in these datasets were aligned using MAFFT v7.221 (Katoh and Toh, 2010), followed by manual alignment to codon position. Specially, the full nucleotide sequences of segments 7 (MP) and 8 (NS) were also aligned using MAFFT v7.221 and the sequences were edited such that all the codons in the first open reading frame (ORF) (M1 or NS1) were followed by the remaining codons in the second ORF (M2 or NEP/NS2) to avoid repetition of nucleotides between the two ORFs. For segments 3 (PA) and 2 (PB1), only the longest ORF was used.

Phylogenetic analyses, correspondence analysis and estimation of codon adaptation index (CAI) were implemented on the datasets of H1 (representing subtypes H1N1 and H1N2), N1, and MP, NS, NP, PA, PB1, PB2 (representing with subtypes H1N1, H1N2, and H3N2). Phylogenetic analyses of all eight gene segments were reconstructed separately with default best-fit models to determine the lineages of these swine influenza viruses using the IQ-TREE (version 1.6.9) package (Nguyen et al., 2015). To improve the tree construction and visualization, two genomes (A/California/04/2009 and A/Canada-ON/RV1527/2009) were chosen to as the representative strains of H1N1/pdm2009 as done in a previous study (Smith et al., 2009).

### Estimating the Codon Usage Patterns of Different Virus Sequences to Host’s Expression System

The human (GenBank Assembly ID: GCA_000001405.28) and swine (GenBank Assembly ID: GCA_000003025) coding sequences were obtained from the Ensembl database^3^. For each host species, all coding sequences were used to calculate the reference Relative Synonymous Codon Usage (RSCU) table, using the program CodonW^4^. Then the CAI value was calculated using the CAIcal web-server^5^ to estimate the adaptability of codon usage patterns of different lineages of SIVs to the host’s expression system. CAI quantifies the similarity of the codon frequency of a set of test sequences (e.g., viral sequences) to those from a reference set of sequences (e.g., host sequence) (Sharp and Li, 1987), which are typically highly expressed host genes. A greater similarity of codon usage of the viral sequences to highly expressed host genes predicts better adaptation of viral genes to their hosts, and higher expression. The index values range from 0 to 1, where the score 1 represents the tendency of a gene to always use the most frequently used synonymous codons in the host (Sharp and Li, 1987).

### Multivariate Analysis

Correspondence analysis (CA) is a type of multivariate statistical analysis that portrays major features of data variation by placing them along continuous axes according to the differential patterns observed, with each consecutive axis having a diminished effect (Clarke, 2007). Each ORF is represented as a 59-dimensional vector and each dimension corresponds to the RSCU value of each codon (all triplets excluding AUG, UGG and stop triplets). Major trends within a dataset can be determined using measures of relative inertia and pertaining data cluster along the different axes of separation according to the variations. CA was performed on the RSCU values using the program CodonW^6^.

### Statistical Analysis

Wilcoxon Rank Sum Test was employed to assess the statistical difference among the CAI values of the each gene segment pertaining to different lineages of SIVs. To investigate the evolution of viral codon usage and associated bias across evolutionary timescale, linear regressions followed by correlation analysis were performed between CAI value and their collection date. The presence of a significant regression coefficient as well as strong correlation (correlation coefficient > 0.4) was considered as supportive of adaptation. All pertaining statistical analysis was performed using SPSS software package (IBM Corp; version 23.0) at 5% level of significance (*p <* 0.05).

## Results

### Identification of SIV Lineages and Reassortment History of the H1N1/pdm2009

A maximum-likelihood phylogenetic inference on 320 strains for each segment, indicated that, the Csw SIVs, EAsw SIVs and TRsw SIVs reflected the majority of the strains circulating in swine population during 1998 to April 2009. As shown in Supplementary Figure S1, the EAsw lineage was the most dominant strain in pigs in European countries, while TRsw lineage with multiple HA and NA types was predominant in pigs in North America. In addition, a set of recombinant human-like H1, with the HA gene derived from human influenza virus and their inner genes similar to TRsw, was found in North America. In Asia, the circulation of SIVs is more complex than it is elsewhere. Apart from these viruses that circulated in North American and European countries, several other lineages have been found only in Asia, such as Csw SIVs and human-origin H3N2 viruses circulating in pigs. Finally, we identified 45, 129, 62, 36, 17, and 31 Csw, EAsw, TRsw, human-like H1, human-origin H3N2 strains and others reassortment strains, respectively, during this period (Supplementary Table S1).

**FIGURE 1:**
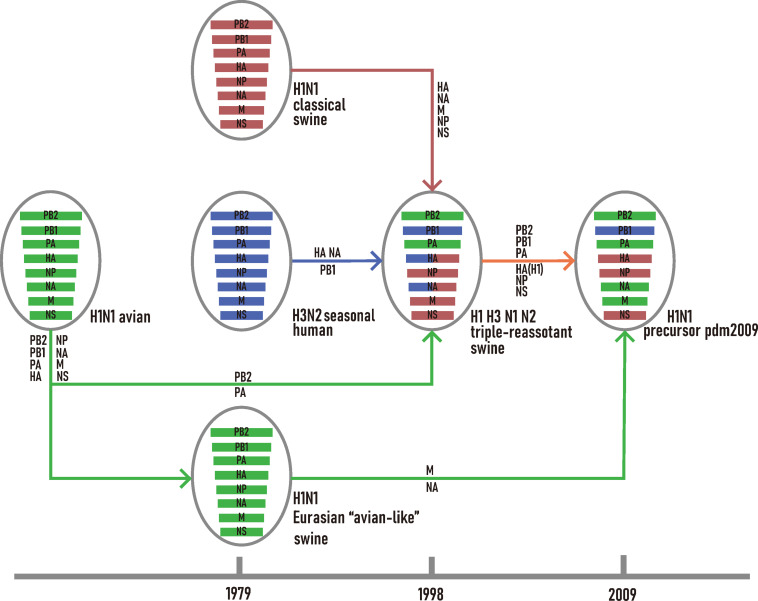
Schematic representation of the genetic reassortant events that led to the development of H1N1/pdm2009. The H1N1 avian lineage, and its descendants are colored in green. The H1N1 classic swine lineage and its descendants are colored in red. The H3N2 seasonal human lineage and its descendants are colored in blue.

For H1N1/pdm2009 virus, its PB2, PB1, PA, HA, NP, and NS were closely related to TRsw SIVs while the MP and NA segments were closely related to EAsw SIVs (Supplementary Figure S1). Combining the outcome of current and previous phylogenetic inferences (Newman et al., 2008; Smith et al., 2009), the reassortment history of H1N1/pdm2009 was reconstructed (Figure 1). All segments of EAsw H1N1 were derived from avian H1N1; the NA, MP genes of H1N1/pdm2009 virus were derived from EAsw H1N1 and the PB2, PB1, PA, HA, NP, and NS of H1N1/pdm2009 virus were derived from TRsw (H1, H3, N1, and N2), which contain the genes M, NP, and NS derived from Csw H1N1, PB2, PA genes directly derived from avian H1N1, PB1 gene derived from human seasonal H3N2, and HA and NA gene derived from Csw H1N1 and human seasonal H3N2.

### Differential Adaptations of Codon Usage Patterns to Human Among Multiple Lineages of SIVs

**FIGURE 2:**
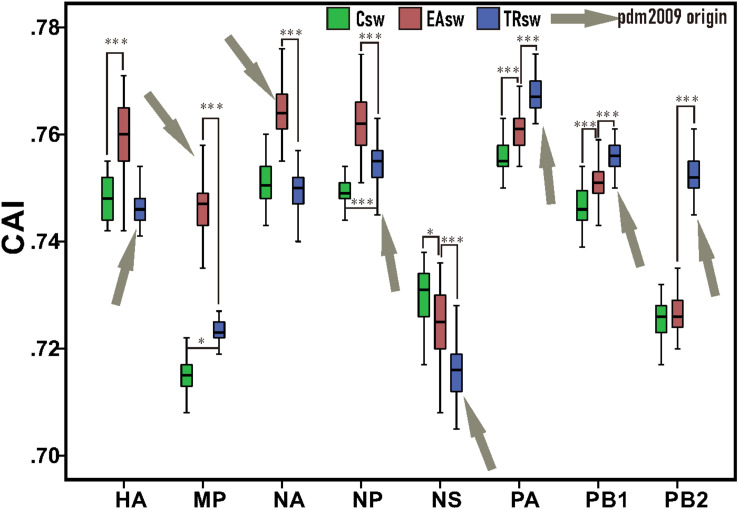
CAI values among each segment belonging to different lineages of SIVs. Green boxes represent values computed from sequences belonging to Csw SIVs and human sequences. Red boxes represent values computed from sequences belonging to EAsw SIVs and human sequences. Blue boxes represent values computed from sequences belonging to TRsw SIVs and human sequences. Arrows marked in gray represent the origin lineage of special gene segment of H1N1/pdm2009. The Wilcoxon Rank Sum Test was used to compare the medians of the CAI values belonging to the different sets of segments of SIVs. ^∗^*p* < 0.05; ^∗∗^ *p* < 0.01; ^∗∗∗^*p* < 0.001.

Our CAI analyses revealed that Csw, EAsw, and TRsw SIVs showed different adaptations to the human codon usage patterns (Figure 2 and Supplementary Table S4). The mean CAI values of the HA, MP, NA, and NP genes from EAsw SIVs were higher (*p* < 0.001) than those of Csw and TRsw SIVs, while the mean CAI values of the PA, PB1, and PB2 genes from TRsw SIVs were the highest. For NS gens, the mean CAI value of NS genes for Csw SIVs was significantly higher than EAsw and TRsw SIVs (*p <* 0.001).

Since the NA and MP genes of H1N1/pdm2009 virus were derived from EAsw SIVs and the PB2, PB1, PA, HA, NP, and NS genes of H1N1/pdm2009 virus were derived from TRsw SIVs (Figure 1), therefore it can be concluded that the H1N1/pdm2009 contains PA, PB1, PB2, NA, and MP segments that were best adapted to the human tRNA pool, while the NS being the least adapted. Notably, similar scenes were showed on the both H1N1/pdm2009 and H3N2, that is, the NS segment showed less adaptation compared with other gene segments, NA showed better adaption than HA, and NP, PA showed better adapted to human codon usage patterns than PB2. However, difference in their adaptation to human codon usage patterns for HA and NA pertaining to H3N2 was obviously showed to be much smaller than that of H1N1/pdm2009 based on our CAI analysis (Figures 2, 4 and Supplementary Figure S2).

### Correspondence Analysis of Codon Usage Variation Among Multiple Lineages of SIVs and Human Host

**FIGURE 3:**
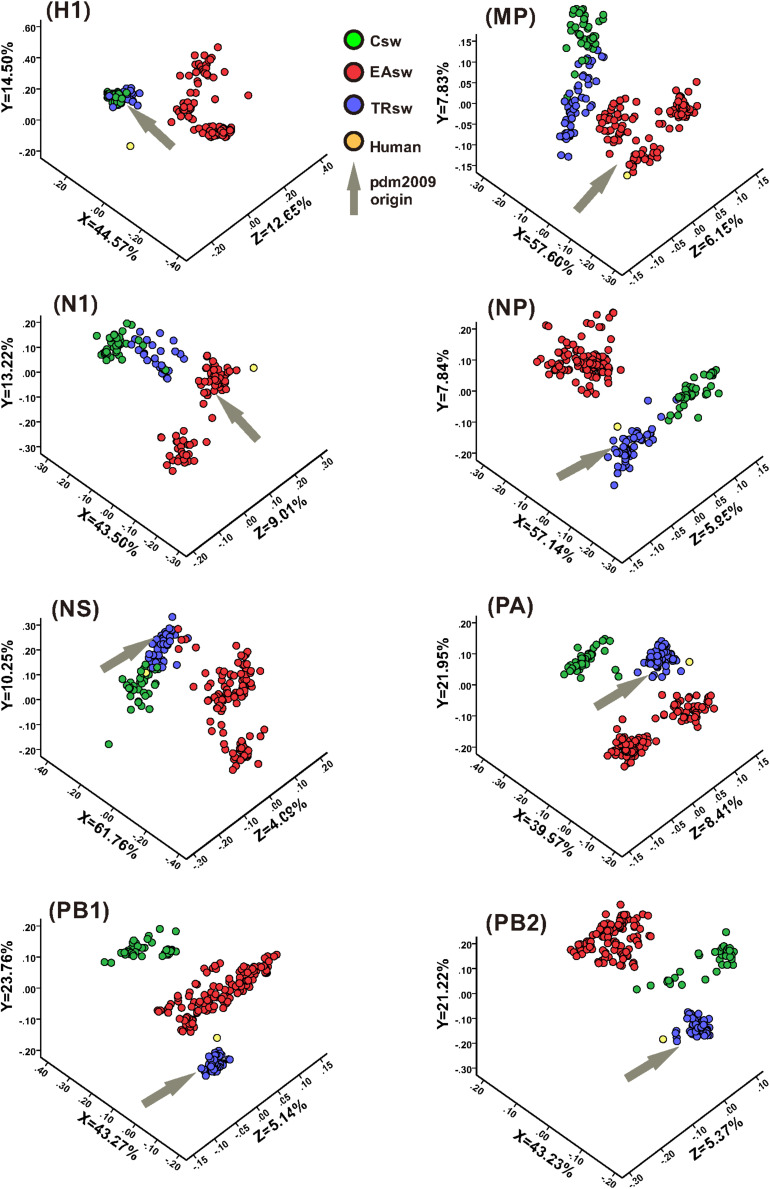
CA analyses of the eight gene segments of various lineages of SIVs. Each viral gene and human sequence are displayed in a three-dimensional representation from a unified perspective. The *X, Y, and Z* axes are in arbitrary scales generated by the CA and the weight of each codon in these axes varies in the different segments. Csw SIVs, EAsw SIVs, and TRsw SIVs are shown in green, red and blue, respectively. The human sequence is shown in yellow and the arrows marked in gray represent the origin lineage of the special gene segment of H1N1/pdm2009.

For large multi-dimensional datasets, CA allows a reduction in the dimensionality of the data to allow visualization to efficiently capture most of the variation and thus provides us with a way to analyze and visualize data (Cvijetic and Djordjevic, 2015). CA was used to further address the codon usage differences among Csw, EAsw, and TRsw SIVs. The RSCU values of the 59 relevant codons were determined for all of the sequences belonging to Csw, EAsw, TRsw SIVs and human sequences, and CA was used on the RSCU values of the different sets of specific lineages as well as specific fragment segments of these three lineages of SIVs and human sequences. The first three major principal axes of separation of data, which account for 65.74–76.09% of the total variations, were used to provide a three-dimensional visualization of the relationships among the sequences from a unified perspective (Figure 3). Generally, different SIVs are located at different positions in the three-dimensional graphs. For HA, MP, NA, NP, and NS segments, the Csw and TRsw SIVs are clustered with each other and separate from Easw SIVs. For PA, PB1, and PB2, these SIVs belonging to various lineages formed discrete clusters.

Human is located closer to TRsw SIVs for the NP, PA, and PB2 segments, while it is located closer to EAsw SIVs for the HA, MP, and NA segments. For PB1, human is located between EAsw and TRsw SIVs, with TRsw SIVs being closer. For the NS segment, human is located closer to Csw SIVs and TRsw SIVs compared with EAsw SIVs.

### Dynamic Evolution on Codon Usage of H1N1/pdm2009 From 2009 to 2019

**FIGURE 4:**
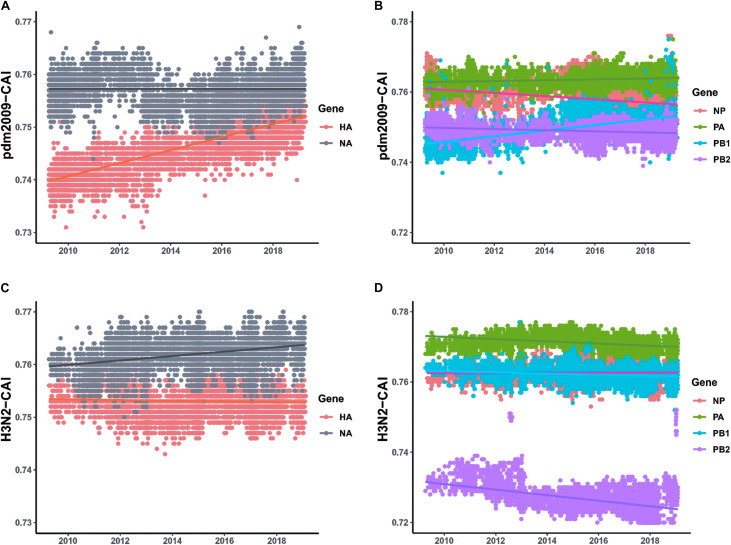
CAI values calculated with respect to human host plotted according to collection date of H1N1/pdm2009 from the April 2009 to April 2019 and the respective regression lines were superimposed using ggplot2 package in R. **(A)** Trends for HA and NA genes pertaining to H1N1/pdm2009. **(B)** Trends for ribonucleoprotein complex encoded genes (NP, PA, PB1, and PB2) pertaining to H1N1/pdm2009. **(C)** Trends for HA and NA genes pertaining to H3N2. **(D)** Trends for ribonucleoprotein complex encoded genes (NP, PA, PB1, and PB2) pertaining to H3N2.

For the two major surface proteins (HA and NA), the mean CAI value of the NA gene is higher than that of the HA gene, and shows relative stability between 2009 and 2019. However, the mean CAI value of the HA segment has shown an upward trend to become close to NA over the last 10 years (correlation coefficient > 0.4, *p <* 0.001) (Figure 4A and Supplementary Table S5). For the ribonucleoprotein complex encoded genes (NP, PA, PB1, and PB2), the mean CAI value of PA and PB2 gene segments has been relatively stable, while the mean CAI value for NP experienced an downward trend (correlation coefficient > 0.4, *p* < 0.001) (Figure 4B and Supplementary Table S5) and the mean CAI value of PB1 experienced a considerable upward trend from 2009 to 2019 (correlation coefficient > 0.4, *p* < 0.001) (Figure 4B and Supplementary Table S5). Generally, the difference of the mean CAI values of these genes, except PA, tends to reduce, indicating an ongoing optimal balance on the fitness to human codon usage pattern among these gene segments. For the MP gene, the mean CAI value across the evolutionary timescale was considerably stable. On the contrary, the NS gene of H1N1/pdm2009 was found to shown a downward trend in the mean CAI value during the phase from 2009 to 2019 (correlation coefficient > 0.4, *p* < 0.001) (Supplementary Figure S2A and Supplementary Table S5). Trends were similar for H1N1/pdm2009 with respect to swine host (Supplementary Figures S2B,C and Supplementary Table S5). For H3N2, NA gene displayed slight ascent trend in mean CAI value (correlation coefficient = 0.357, *p* < 0.05), while PB2 gene showed a significant downward trend across the timescale from 2009 to 2019 (correlation coefficient > 0.4, *p* < 0.001). All other gene segments pertaining to H3N2 were noted to be relatively stable (Supplementary Table S5).

## Discussion

Due to its unique feature that harbor receptors for both avian-adapted and mammal-adapted influenza virus, swine are thought to be intermediate “mixing vessel” where both avian and human influenza viruses can undergo genetic reassortment, showing potential to generate novel viruses that cause pandemic in human (Scholtissek, 1990).

This potential has been borne out with the emergence H1N1/pdm2009, which is derived from multiple reassortant events among several lineages of coexisting swine influenza viruses (Figure 1). In this study, we found that the PB2, PA, PB1 genes derived from TRsw SIVs and the NA and M genes derived from EAsw SIVs, which were converged to develop the precursor of H1N1/pdm2009, showed a higher adaptability to the codon usage pattern of human compared with the same segments of other lineages of SIVs. All these genes encode proteins that play an important role in the viral reproduction cycle. Replication and transcription of influenza virus are catalyzed by the viral polymerase complex, which is composed of the PB2, PB1, and PA proteins (Pflug et al., 2017). The main function of NA protein is as a sialidase to cleave sialic acid from cell surfaces during the final stages of the replication cycle, enabling the release of virion progeny (Palese et al., 1974; Suzuki et al., 2005). M1 protein binds to the vRNP complex and NEP/NS2 protein, while M2 protein acts as an ion channel, and collectively they mediate the process of vRNP complex export from the nucleus (Boulo et al., 2007; Rossman et al., 2010; Wang et al., 2012). Thus, better adaptation these sequences to host tRNA pool should help boost the replication of H1N1/pdm2009. In fact, the avian-origin PB1 gene segment, which is the initial source for both H3N2/1968 and H1N1/pdm2009, have been shown to enhance viral growth and transmissibility, likely by enhancing activity of the viral polymerase complex (Wendel et al., 2015). Such an observation has been consistent with its high adaptation to the human tRNA pool based on our CAI analysis in this study. Besides, during the emergence of H7N9, a similar preference to using polymerase and nucleoprotein genes of H9N2 that better fit the codon usage of chicken was discovered (Luo et al., 2019a).

After the emergence of a novel virus, the subsequent spread within a new host population requires a period of adaptation of the virus to the new host (Webster et al., 1992). For example, modern H3N2 viruses changed their codon usage to better reflect tRNA availabilities in IFN-treated cells, to be more efficiently translated than their ancestors from 1968 (Smith et al., 2018). In this study, compared with contemporaneous human seasonal influenza virus H3N2, which show much stable host adaption, we found that the codon usage of the H1N1/pdm2009 viruses drastically adjust their codon usage pattern, indicating great pressure to accumulate genetic changes to further hone their acclimatization with human synthetic machinery (Figure 4 and Supplementary Figure S2). H1N1/pdm2009 has been introduced at a much later stage than H3N2 and still exhibit patterns of ongoing adaptation. Interestingly, when we focused on the evolutionary patterns of host adaption for both the H1N1/pdm2009 and human seasonal influenza virus H3N2 with respect to swine hosts, which is considered to be the ultimate origin of H1N1/pdm2009 and has experienced a large-scale reverse zoonosis of human seasonal influenza viruses (Nelson and Vincent, 2015), a similar evolutionary scene was revealed between human and swine hosts (Figure 4 and Supplementary Figure S2). A reasonable explanation is that, human beings and swine are two closely related species and common tissue specific expression patterns have been established between the two species (Hornshoj et al., 2007). Thus, it is predictable that the viruses have undergone evolutionary changes targeted at enhanced fitness and adaptation to the expression system of both the hosts.

A thorough estimation of the CAI values of the gene segments of H1N1/pdm2009, calculated with respect to human, revealed that the codon usage of different genes of H1N1/pdm2009 tended to gradually optimal balance during the period 2009 to 2019. A steady rise in adaption of HA gene in comparison to relatively stable adaptation of NA gene pointed toward a tendency of reducing the difference in CAI values and thus attaining an optimal balance for adaption to human cellular system for this two gene segments. A functional optimal balance between the activities of HA and NA is required for efficient viral replication and transmission (Mitnaul et al., 2000; Yen et al., 2011). Interestingly, similar propensity of achieving an optimal balance in CAI values was also noted among ribonucleoprotein complex genes (Figure 4 and Supplementary Table S5). The polymerase proteins (PB2, PB1, and PA) together with NP protein encapsulate the viral RNA to form the ribonucleoprotein complex (RNP), which is the minimal functional unit of the viral genome for transcription and replication (Pleschka et al., 1996; Neumann et al., 1999). During the process of virus replication, the RNP complex containing the polymerase proteins and NP protein synchronously access, or export from, the nucleus of host cells (Chou et al., 2013). PB2, PA, and NP proteins often co-evolve within strains, most likely as a result of the important physical and functional interactions these proteins have with each other (Obenauer et al., 2006; Naffakh et al., 2008). Thus, incompatibility of any protein of reassortant-vRNPs will affect the overall RNP production and thus the replication rate of the whole virus. It has been reported that the presence and distribution of preferred and disfavored codons has been suggested as a factor guiding the proper protein folding (Ran and Higgs, 2012). Presumably, the ongoing optimal balance in adaptation in human cellular environment among these genes might be a strategy to regulate the protein production and the protein spatial structure, facilitating better protein–protein interactions that in turn affect viral transcription, replication and viral ribonucleoprotein assembly and HA/NA balance at the level of protein function. However, functional experiments are needed to further support this hypothesis.

Notably, the HA, NP, and NS genes of H1N1/pdm2009 precursors show less adaptation to the codon usage pattern of human (Figure 2), and the NS gene was noted to further reduce its similarities in codon usage patterns with that of human based on our CAI analysis (Supplementary Figure S2A). An explanation for this opposite trend is that, host immune response is a key factor restricting virus cross-species transmission, and antagonistic portions of codon usage pattern between a virus and its host may reduce the stimulation of the host immune system and thus contribute to immune evasion by the virus (Moratorio et al., 2013), a strategy reported in the host adaptation of the Epstein-Barr virus (Karlin et al., 1990). In addition, the formation and propagation of reassortant viruses are subject to a complex array of determining factors that involve the compatibility of packaging signals and proteins interactions (Lowen, 2017), which may obscure the impact of the selective advantage for the adaptability of codon usage. Furthermore, one limitation of the present research is that the host adaptation of H1N1/pdm2009 in special cases, such as drug treatment or host autoimmune rejection, where the tRNA pool is expected to be altered compared to the normal conditions, has not been taken into consideration (Gingold et al., 2014). It is possible that this regulation in the codon usage and de-optimal trend in special genes would lead to increased fitness in certain circumstances in a way that still remains unaddressed, as in the case of modern H3N2 (Smith et al., 2018). Further studies are necessary to arrive at a final inference.

## Conclusion

The codon usage perspective suggests that the build-up of the gene cassettes of advantageous genes that boost viral replication should be a favorable factor that contributes to the development of H1N1/pdm2009. This strategy benefits its efficient replication and strengthens its competitiveness. After its emergence, an ongoing optimal balance for its genes has been a major selective force that boosted the evolution of its codon usage to further better the fit to the human host.

## Data Availability Statement

Publicly available datasets were analyzed in this study. The accession numbers can be found in Supplementary Tables S1–S3.

## Ethics Statement

We used publicly available sequence data from NCBI and GISAID. No ethical consideration is required.

## Author Contributions

YS conceived, designed, and supervised the study. FG, JY, JP, XL, and XS collected and analyzed the data. YS and DI wrote the drafts of the manuscript. R-AC commented on and revised the drafts of the manuscript. All the authors read and approved the final draft of the manuscript.

## Conflict of Interest

R-AC was employed by Guangdong Enterprise Key Laboratory of Biotechnology R&D of Veterinary Biological Products. The remaining authors declare that the research was conducted in the absence of any commercial or financial relationships that could be construed as a potential conflict of interest.

## Footnotes

1 www.ncbi.nlm.nih.gov/genomes/FLU

2 www.gisaid.org

3 http://www.ensembl.org

4 http://sourceforge.net/projects/codonw

5 http://genomes.urv.es/CAIcal/

6 http://sourceforge.net/projects/codonw/

## Supplementary Material

FIGURE S1 Phylogenetic trees for each gene segment pertaining to SIVs collected between 1998 and April 2009 with two representative H1N1/pdm2009 strains (A/California/04/2009 and A/Canada-ON/RV1527/2009). These sequences are classified into different lineages according to the tree topology and bootstrap values of >80% (outer circle). The classifications of separated regions (location) and subtypes have been shown in the middle circle and inner circle respectively. Arrows in hollow represent the location of two representative H1N1/pdm2009 strains in phylogenetic tree.

FIGURE S2 CAI values plotted according to collection date from April 2009 to April 2019 and the respective regression lines were superimposed using ggplot2 package in R **(A)** Trends for MP and NS gene for H1N1/pdm2009 (top) and H3N2 (bottom), with respect to human host. **(B)** Trends for MP and NS gene for H1N1/pdm2009 (top) and H3N2 (bottom), with respect to swine host. **(C)** Trends for HA, NA, NP, PA, PB1 and PB2 genes for H1N1/pdm2009 (top) and H3N2 (bottom), with respect to swine host.

TABLE S1 Strain information, and CAI values of 320 complete SIV genomes collected between 1998 and April 2009.

TABLE S2 Strain information, and CAI values of H1N1/pdm2009 viruses collected from April 2009 to April 2019.

TABLE S3 Strain information, and CAI values of H3N2 viruses collected from April 2009 to April 2019.

TABLE S4 The mean CAI values and statistical analysis of eight genes from three lineages of SIVs.

TABLE S5 Line of fit and correlation analysis (Spearman’s rank correlation) of CAI values (calculated with respect to human and swine host) with collection date for each gene segment pertaining to H1N1/pdm2009 and H3N2.
