# Supplementary material for: Origin and Evolution of H1N1/pdm2009: A Codon Usage Perspective
Source: Front Microbiol. 2020 Jul 14;11:1615. doi: 10.3389/fmicb.2020.01615 (PMC7372903; doi:10.3389/fmicb.2020.01615)
Supplement: FIGURE S1 — Phylogenetic trees for each gene segment pertaining to SIVs collected between 1998 and April 2009 with two representative H1N1/pdm2009 strains (A/California/04/2009 and A/Canada-ON/RV1527/2009). These sequences are classified into different lineages according to the tree topology and bootstrap values of >80% (outer circle). The classifications of separated regions (location) and subtypes have been shown in the middle circle and inner circle respectively. Arrows in hollow represent the location of two representative H1N1/pdm2009 strains in phylogenetic tree. [file Data_Sheet_1.PDF]

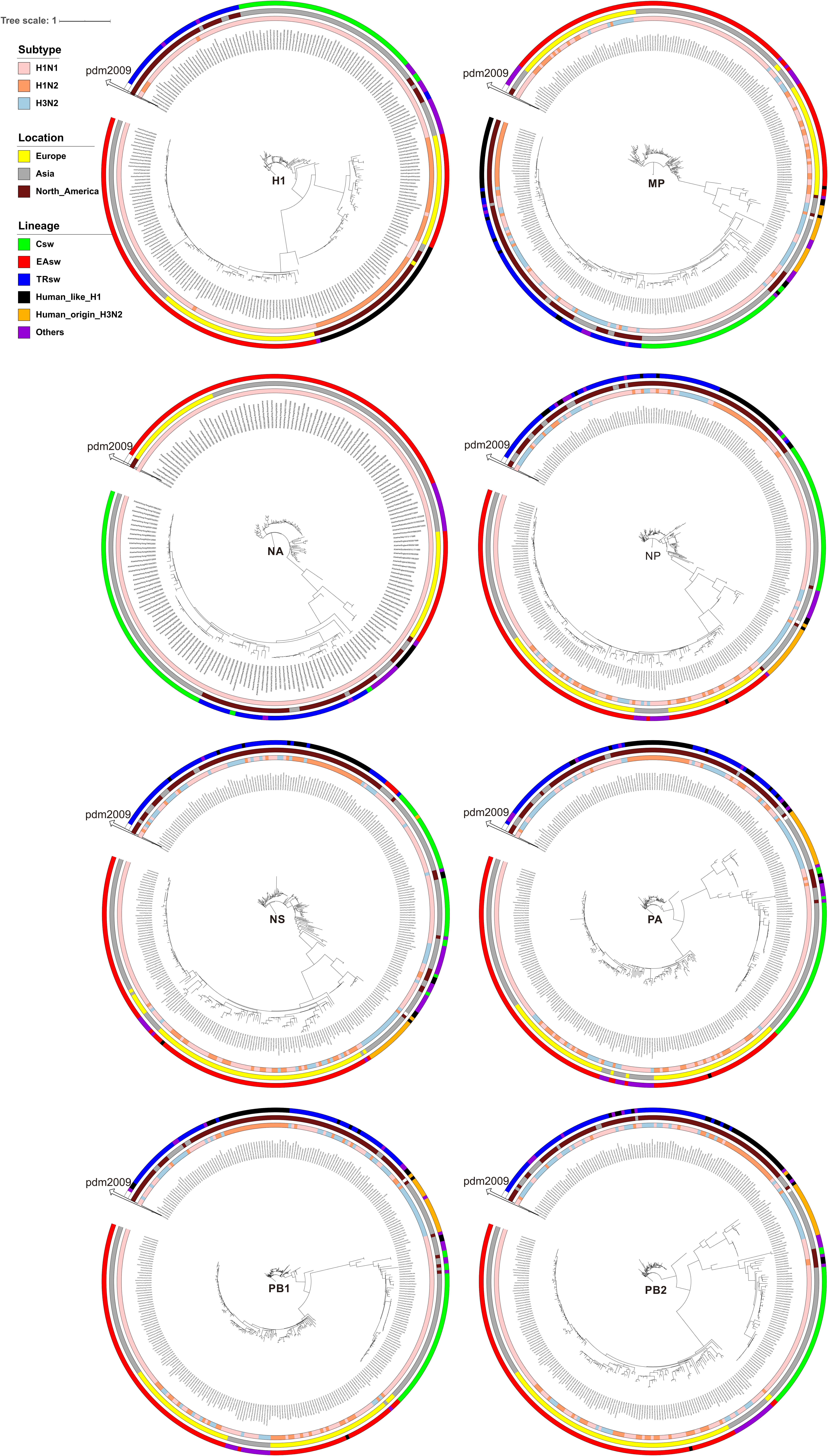
Tree scale: 1
Subtype
H1N1
H1N2
H3N2
Location
Europe
Asia
North_America
Lineage
Csw
EAsw
TRsw
Human_like_H1
Human_origin_H3N2
Others
pdm2009
H1
MP
NA
NP
NS
PA
PB1
PB2
