# Supplementary figures and images for: Origin and Evolution of H1N1/pdm2009: A Codon Usage Perspective
Source: Front Microbiol. 2020 Jul 14;11:1615. doi: 10.3389/fmicb.2020.01615 (PMC7372903; doi:10.3389/fmicb.2020.01615)

(A)

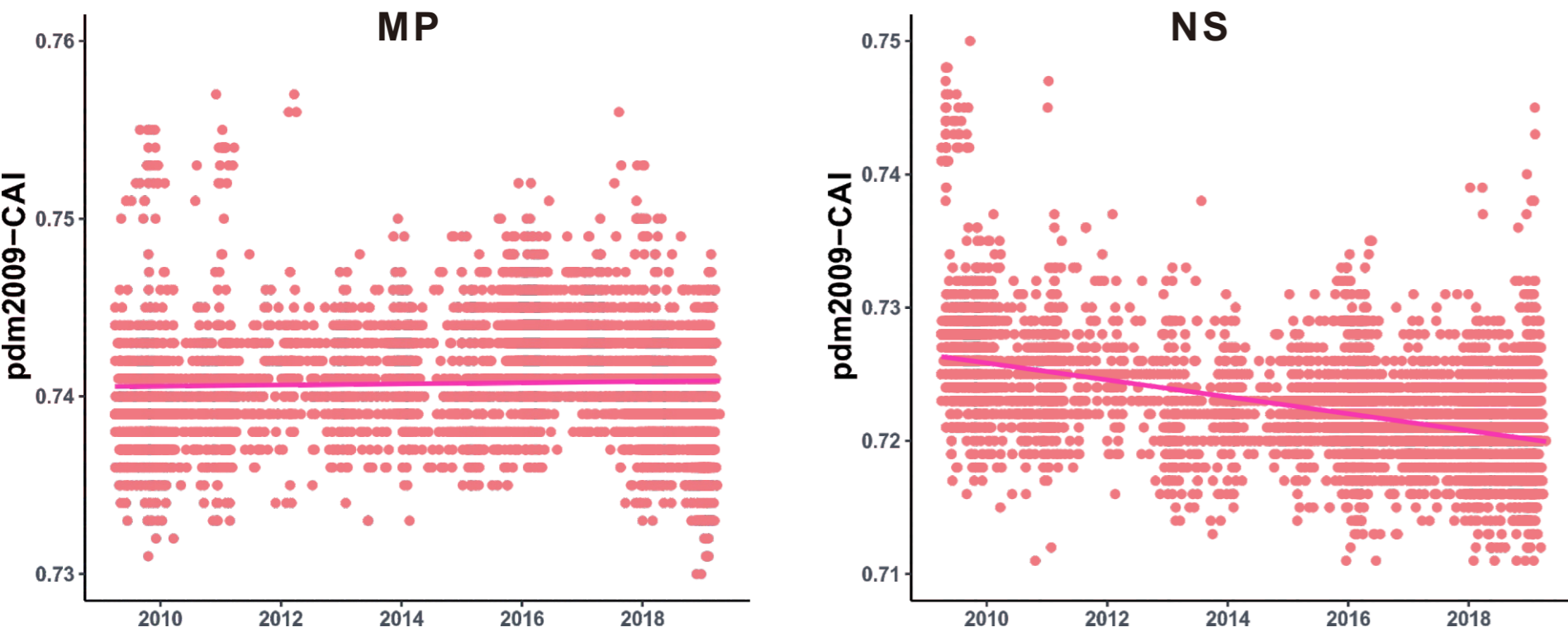

(B)

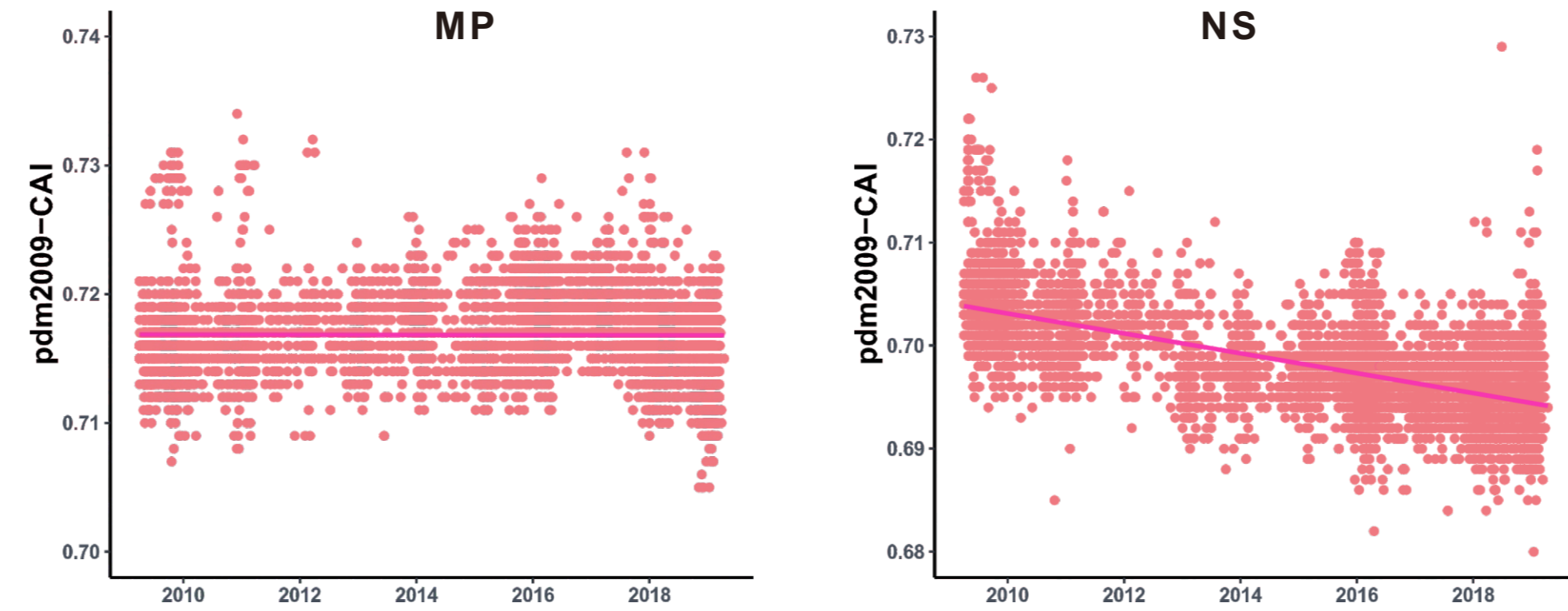

(C)

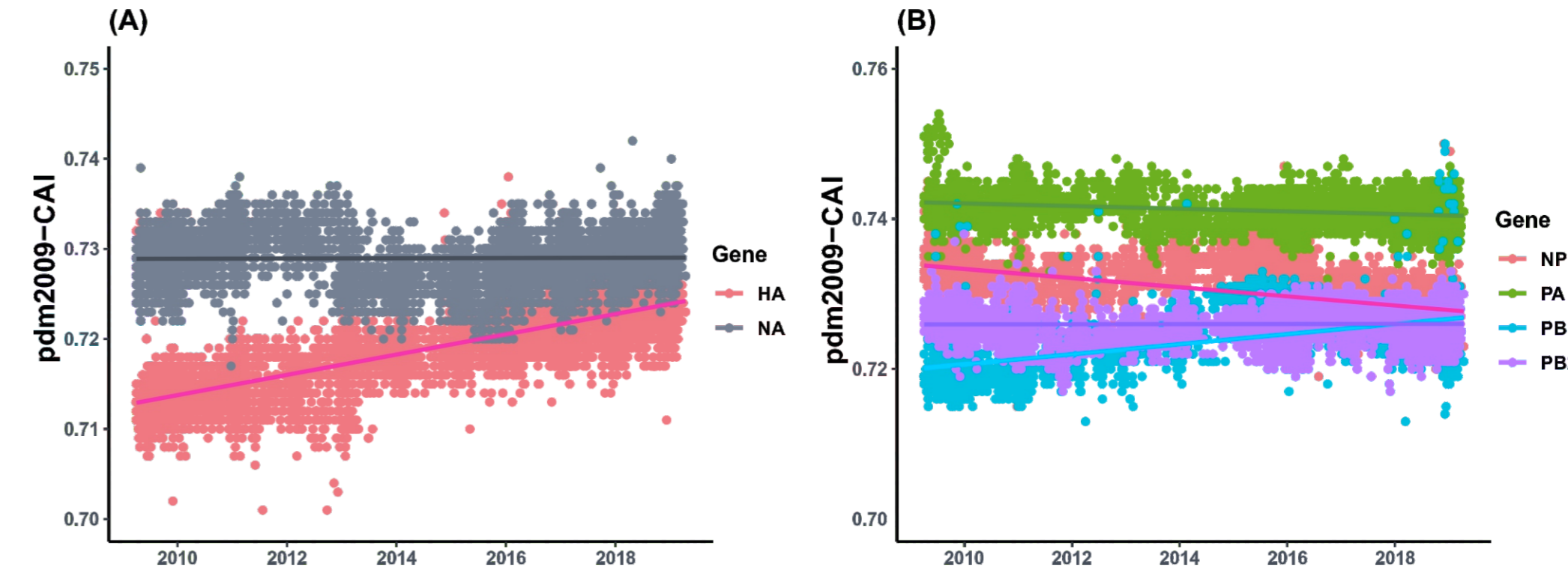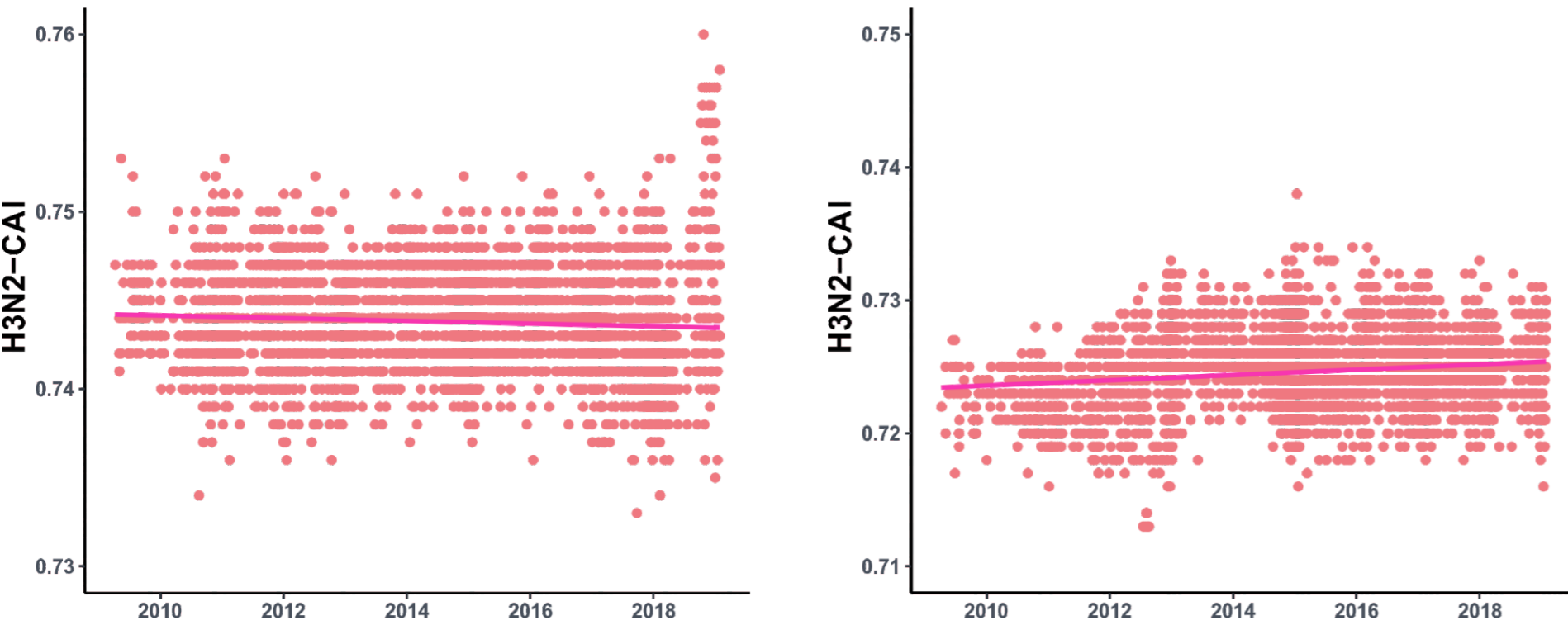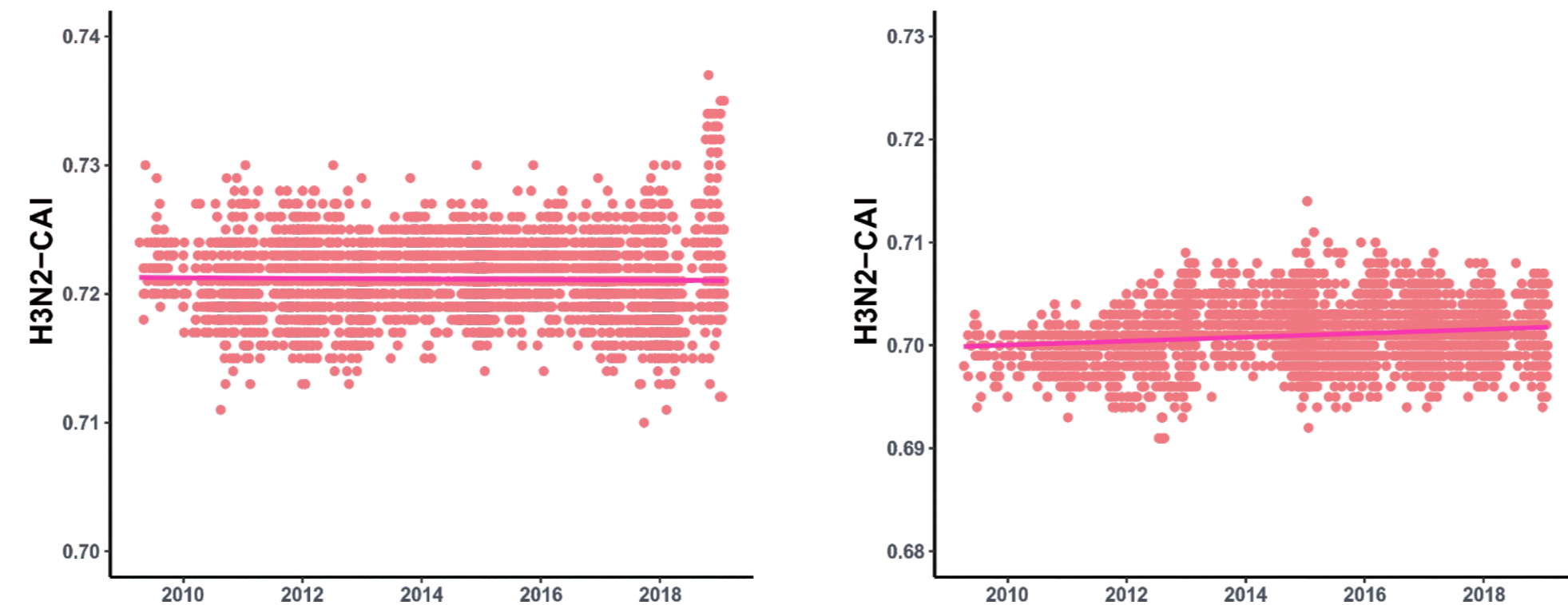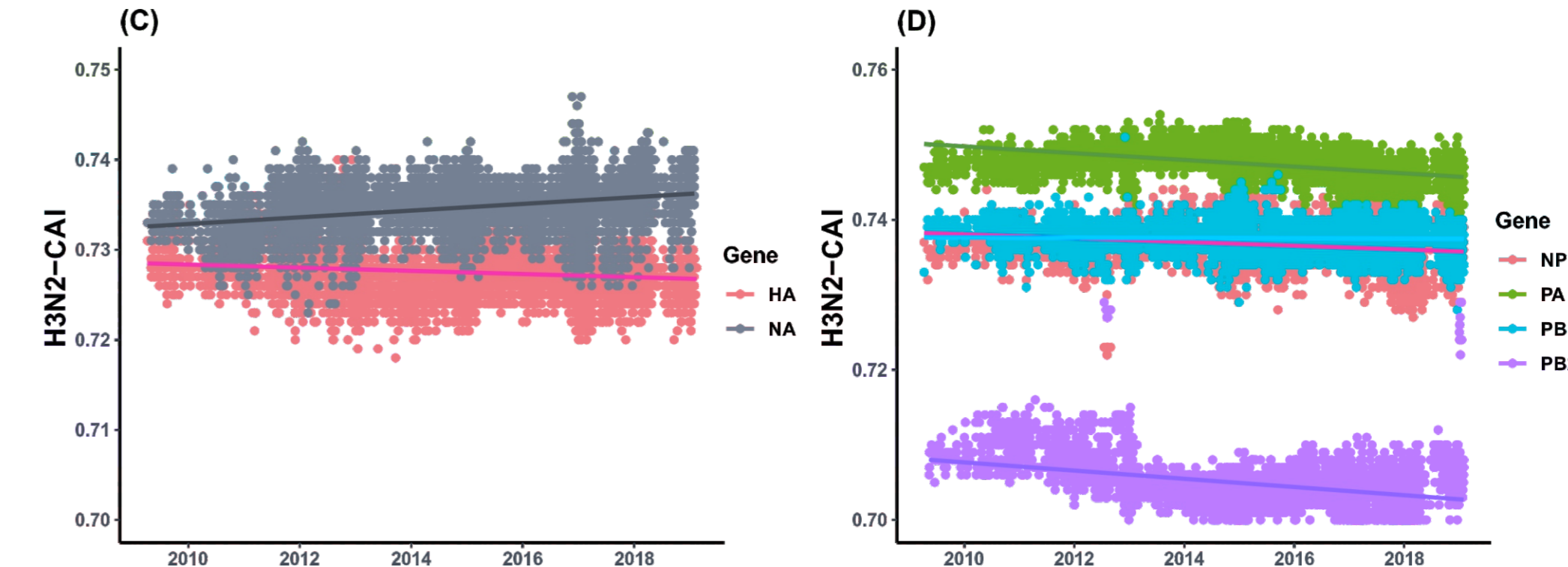

Supplement: FIGURE S2 — CAI values plotted according to collection date from April 2009 to April 2019 and the respective regression lines were superimposed using ggplot2 package in R (A) Trends for MP and NS gene for H1N1/pdm2009 (top) and H3N2 (bottom), with respect to human host. (B) Trends for MP and NS gene for H1N1/pdm2009 (top) and H3N2 (bottom), with respect to swine host. (C) Trends for HA, NA, NP, PA, PB1 and PB2 genes for H1N1/pdm2009 (top) and H3N2 (bottom), with respect to swine host. [file Data_Sheet_2.PDF]
