# Supplementary material for: Origin and Evolution of H1N1/pdm2009: A Codon Usage Perspective
Source: Front Microbiol. 2020 Jul 14;11:1615. doi: 10.3389/fmicb.2020.01615 (PMC7372903; doi:10.3389/fmicb.2020.01615)
Supplement: TABLE S5 — Line of fit and correlation analysis (Spearman’s rank correlation) of CAI values (calculated with respect to human and swine host) with collection date for each gene segment pertaining to H1N1/pdm2009 and H3N2. [file Table_5.DOCX]

|  |  | CAI-Human^a^ | | CAI-Swine^b^ | |
| --- | --- | --- | --- | --- | --- |
| Subtype | Gene | Fit line | Correlation coefficient (r) | Fit line | correlation |
| Pdm2009 | HA | Y=1.74+1.23E-3*X | 0.872^**^ | Y=1.55+1.13E-3*X | 0.862^**^ |
|  | MP | Y=0.68+3.17E-5*X | 0.036^**^ | Y=0.73-5.51E-6*X | ns |
|  | NA | Y=0.76-8.36E-7*X | ns | Y=0.7+1.27E-5*X | ns |
|  | NP | Y=1.67-4.51E-4*X | 0.475^**^ | Y=1.95-6.08E-4*X | 0.590^**^ |
|  | NS | Y=2.01-6.37E-4*X | 0.555^**^ | Y=2.64-9.65E-4*X | 0.725^**^ |
|  | PA | Y=0.53+1.18E-4*X | 0.204^**^ | Y=1.1-1.78E-4*X | 0.339^**^ |
|  | PB1 | Y=0.88+8.09E-4*X | 0.687^**^ | Y=0.65+6.8E-4*X | 0.632^**^ |
|  | PB2 | Y=1.09-1.7E-4*X | 0.187^**^ | Y=0.73+6.24E-8*X | ns |
| H3N2 | HA | Y=0.89-6.73E-5*X | 0.065^**^ | Y=1.08-1.75E-4*X | 0.181^**^ |
|  | MP | Y=0.9-7.66E-5*X | 0.067^**^ | Y=0.77-2.45E-5*X | ns |
|  | NA | Y=-0.09+4.22E-4*X | 0.357^**^ | Y=-0.01+3.72E-4*X | 0.344^**^ |
|  | NP | Y=0.72+2.13E-5*X | ns | Y=1.24-2.51E-4*X | 0.201^**^ |
|  | NS | Y=0.33+1.98E-4*X | 0.156^**^ | Y=0.32+1.91E-4*X | 0.155^**^ |
|  | PA | Y=1.41-3.16E-4*X | 0.356^**^ | Y=1.65-4.48E-4*X | 0.478^**^ |
|  | PB1 | Y=1.06-1.49E-4*X | 0.168^**^ | Y=0.78-2.09E-5*X | 0.026^*^ |
|  | PB2 | Y=2.6-9.31E-4*X | 0.549** | Y=2.43-8.55E-4*X | 0.531** |

Supplementary Table 5. Line of fit and correlation analysis (Spearman’s rank correlation) of CAI values (calculated with respect to human and swine host) with collection date for each gene segment pertaining to pdm2009/H1N1 and H3N2.

**-Correlation is significant at the 0.01 level (2-tailed); *-Correlation is significant at the 0.05 level (2-tailed);a-CAI value calculated with respect to human host; b-CAI value calculated with respect to swine host; ns-no significant; Yellow background- Gene segments with high correlation between collection date and CAI value(r>0.4).
